# The Year-Book of Treatment

**Published:** 1890-03

**Authors:** 


*The Year-book of Treatment.* (CASSELL & Co., London.)
*The Medical Annual.* (WRIGHT & Co., Bristol.)
*Braithwaite's Retrospect of Medicine.*

These admirable compendiums come to hand with fair punctuality. *The Medical Annual* maintains its excellence as an impersonal and trustworthy record of scientific and therapeutic progress. Cassell's *Year-book*, full and complete and thorough and highly scientific as it is, continues to be here and there disfigured by personal opinions and comments of the abstractors, which we could always dispense with and would often contradict. *Braithwaite* is such an old and valued and trustworthy friend, that his welcome may be taken for granted.

